# "In Hot Haste," etc.

**Published:** 1888-09-01

**Authors:** 


### Turning Over New Leaves.

[Books for Review should be sent to The Editor, The Lodge, Porchester Square, W.]

### A Tale of German Life.*

Novels dealing with the misunderstandings of youths and maidens who marry in haste and repent at leisure are common enough, and are usually painful, but Miss Hullah's book, though it has plenty of pathos, is kind enough to leave us at the last not merely with the satisfactory knowledge that Kurt Weide and his wife, Sabine, are reconciled, but that throughout all their errors and misunderstandings they are worthy of our respect. The pictures of German life and character are admirably done, while Sabine's sufferings when she is governess in a snobbish English family, who are afraid that they will derogate from their position if they treat her with decent courtesy, are related with a freshness and humour that do not, however, conceal the underlying sad truth of the situation.

### The Student's Handbook of Chemistry.†

To a certain class of students who have had no scientific teaching at school, the study of chemistry presents peculiar difficulties. It is, therefore, of extreme importance that elementary text-books, prepared for their use, should be as plain, precise, and simple as possible. They should, moreover, deal only with essential general principles and facts of real importance. Historical and other unessential details are out of place in such books, and should be rigorously excluded. The little work written by Mr. Leicester Greville, and published by Messrs. E. and S. Livingstone, the well-known medical publishers of Edinburgh, fulfils these necessary conditions very well. It is simple and precise, and deals intelligently with the difficulties of the learner. It is quite on the lines of the ordinary text-books, but excels some of them in its greater simplicity and felicity of expression. The work is divided into Inorganic and Organic Chemistry. There is also a separate section on Animal Chemistry, which will be found very convenient for medical students. An additional merit is the presence of several pages of definitions, a much-needed feature of all text-books dealing with sciences previously unfamiliar to the learner. The price is not given, but as the work is small the cost cannot be great. As a book for students it may be commended for its brevity, simplicity, and fullness. There is a misprint on p. 10, which ought, in the interests of beginners, to be corrected without delay.

* "In Hot Haste." By Mary E. Hullah. Richard Bentley and Sons. Two vols.

† By H. Leicester Greville, F.I.C., F.C.S., &c. Edinburgh: E and S. Livingstone.

